# The effect of participant nonresponse on HIV prevalence estimates in a population-based survey in two informal settlements in Nairobi city

**DOI:** 10.1186/1478-7954-8-22

**Published:** 2010-07-22

**Authors:** Abdhalah K Ziraba, Nyovani J Madise, Mwau Matilu, Eliya Zulu, John Kebaso, Samoel Khamadi, Vincent Okoth, Alex C Ezeh

**Affiliations:** 1 African Population and Health Research Center, Shelter Afrique Centre, 2nd Floor, Longonot Road, Upper Hill P. O. Box 10787, 00100, Nairobi Kenya; 2 Department of Epidemiology and Population Health, Centre for Population Studies. London School of Hygiene and Tropical Medicine. 49-51 Bedford Square, London, WC1B 3DP, UK; 3 University of Southampton, School of Social Sciences, Southampton, SO17 1BJ, UK; 4 Kenya Medical Research Institute (KEMRI). P. O. Box 54840, 00200, Nairobi Kenya; 5 Loma Linda University, School of Public Health, 24951 North Circle Drive, Nichol Hall 1410 Loma Linda, California 92350, USA

## Abstract

**Background:**

Participant nonresponse in an HIV serosurvey can affect estimates of HIV prevalence. Nonresponse can arise from a participant's refusal to provide a blood sample or the failure to trace a sampled individual. In a serosurvey conducted by the African Population and Health Research Center and Kenya Medical Research Centre in the slums of Nairobi, 43% of sampled individuals did not provide a blood sample. This paper describes selective participation in the serosurvey and estimates bias in HIV prevalence figures.

**Methods:**

The paper uses data derived from an HIV serosurvey nested in an on-going demographic surveillance system. Nonresponse was assessed using logistic regression and multiple imputation methods to impute missing data for HIV status using a set of common variables available for all sampled participants.

**Results:**

Age, residence, high mobility, wealth, and ethnicity were independent predictors of a sampled individual not being contacted. Individuals aged 30-34 years, females, individuals from the Kikuyu and Kamba ethnicity, married participants, and residents of Viwandani were all less likely to accept HIV testing when contacted. Although men were less likely to be contacted, those found were more willing to be tested compared to females. The overall observed HIV prevalence was overestimated by 2%. The observed prevalence for male participants was underestimated by about 1% and that for females was overestimated by 3%. These differences were small and did not affect the overall estimate substantially as the observed estimates fell within the confidence limits of the corrected prevalence estimate.

**Conclusions:**

Nonresponse in the HIV serosurvey in the two informal settlements was high, however, the effect on overall prevalence estimate was minimal.

## Background

Selective participation in a study can potentially skew estimates of the outcome of interest in a study population [1-5]. This is more likely to be the case if the circumstances that influence low participation are in some way related to the main outcome. Nonresponse in HIV serosurveys is mainly due to refusal to provide a blood sample for HIV testing or absenteeism of the sampled individual during the survey period. Several population-based HIV seroprevalence studies have reported varying nonresponse rates for HIV testing, ranging from as low as 5% among men in Rwanda to 56% in Lesotho [2,3]. A moderate nonresponse rate (14.4% ) for HIV testing for Kenya was reported in an earlier survey[2]. From the studies carried out to date on this topic, it has been shown that the effect of participant nonresponse on HIV prevalence estimates vary by certain characteristics, such as gender and residence, among others. Yet in general, the overall effect on national estimates is small, unless the level of nonresponse is very high, as was the case in Lesotho [1-3,6].

HIV/AIDS remains a highly stigmatized disease, with many people preferring either not to know their status or to keep it a secret [7,8]. The preference of an individual not to participate in a serosurvey may partly be influenced by the fear of knowing his or her own HIV serostatus. On the other hand, those who know their status as positive may participate in a serosurvey in the hope that they can be helped, or they may choose not to participate as they see no immediate benefit. Personal perceived risk may be correlated with actual risk of HIV infection [9]. Perceptions about HIV risk are unlikely to be random among individuals in a population; they are likely to vary by defined individual characteristics, such as race, religion, ethnicity, and past behaviors, including experience with drug use or sex work [10]. For that reason, if participants who perceive themselves to be at a higher risk of contracting HIV do not participate in a serosurvey, then prevalence estimates may be biased downward and might affect the overall estimate.

Interviewers may fail to make contact with a sampled person for a number of reasons, including temporary absence, work patterns, inability to locate the household/structure in which the sampled person lives, and out-migration. Highly mobile individuals, such as long-distance truck drivers, security personnel, and migrant workers, often have a different level of exposure to the risk of HIV [11-14]. In highly mobile populations, many sampled individuals may not be contacted, even if a good random sample is drawn. If a population has a substantial proportion of highly mobile individuals who miss out on a seroprevalence study and yet are likely to be at a higher risk, the estimates are likely to be biased downward as less mobile and low risk individuals are overrepresented in the effective sample interviewed [2,3]. On the other hand, if a majority of a community's residents are migrant workers who live away from their families, they are likely to be exposed to higher risks of HIV infection. To the extent that such individuals are overrepresented in a seroprevalence survey, estimates are likely to be biased upward.

### The slum context

Although informal settlements in Nairobi city are home to more than 60% of Nairobi's population [15], the informal nature of housing is likely to lead to underrepresentation of the slum population in national surveys, given the difficulty involved in listing temporary housing structures. Until the project on which this paper is based was conducted, HIV prevalence in the informal settlements was unknown. Kenya has had at least two large population-based HIV testing surveys [16,17]. The Kenya Demographic and Health Survey of 2003 put the HIV prevalence estimate for Nairobi province at 10%. Nyanza province had the highest prevalence rate at 15%, and the national prevalence rate was 6.7% [16]. There were differences in HIV prevalence rates by age, gender, ethnicity, rural-urban residence, educational attainment, and wealth status. These differences have been observed in several other surveys in sub-Saharan countries [2,3,16]. A more recent survey, the Kenya AIDS Indicator Survey 2007, estimated the national prevalence to be 7% and Nairobi province's prevalence rate to be 9% [17]. However, the national surveys are unable to provide HIV prevalence estimates for slums. Earlier behavioral research indicates that high-risk sexual practices are prevalent in the informal settlements of Nairobi [18,19]. Furthermore, recent work using verbal autopsies to establish causes of death, without HIV status, showed that HIV/AIDS and tuberculosis accounted for more than 50% of the adult mortality burden in the slums [20].

The African Population and Health Research Center (APHRC), in partnership with the Kenya Medical Research Institute (KEMRI), carried out a survey to estimate the prevalence and risk factors for HIV in two informal settlements in Nairobi city. The two communities where the project was carried out are informal settlements characterized by poor housing, lack of clean water, poor sanitation, unemployment, poverty, and overcrowding. Viwandani slum is located very close to the city's industrial area and is home to many low-income youths working in the industries close by. Korogocho is a more established slum settlement with a high proportion of men living with their spouses and children. Korogocho residents are predominantly either very low-income earners or unemployed. Additionally, residents of Viwandani are relatively more educated than those of Korogocho.

The survey, like many community-based surveys, faced a challenge of nonresponse, with a sizeable proportion of sampled individuals being nonresponders (43%). The desire to understand the effect of nonresponse on prevalence estimates was the basis for this paper. We hypothesised that the HIV prevalence estimate in the survey was underestimated due to low participation of highly mobile community members. Specifically, this paper aimed to describe selective participation in the serosurvey by sociodemographic characteristics and also to estimate the bias in the estimates of HIV prevalence.

## Methodology

Data used in this paper came from a cross-sectional serosurvey carried out from September 2006 to November 2007. The project was nested in the Nairobi Urban Health and Demographic Surveillance System (NUHDSS) covering about 60,000 individuals in two slums: Korogocho and Viwandani. The NUHDSS database provided the sampling frame from which a random sample of eligible participants was drawn. Eligible individuals had to be residents in the demographic surveillance area, registered with the NUHDSS, and aged between 15 to 54 years for men and 15 to 49 years for females. A total of 5,004 individuals were sampled. However, after the study and with the benefit of extra DSS updates of the residency status of individuals under surveillance, 237 individuals were found to have not been legitimate residents at the time the sample was drawn. These individuals have thus been excluded from the overall sample, leaving a total of 4,767.

A list of all sampled participants was generated with enough information to enable field workers to positively identify participants in their households. On the other hand, the questionnaires and blood sample filter papers didn't contain any identifiers except a new identification number (ID) to allow linkage to the NUHDSS data. A minimum of three visits were made for individuals who were not found at home on the first visit, and security arrangements were made to interview individuals who were identified as only available at odd hours (very early in the morning or late in the evening).

Participants were given information about the objectives of the study and information about their rights. Potential risks and benefits were read aloud by the interviewer to those who could not read, and those who could read were allowed enough time to read before making a decision. Those who accepted to participate affirmed it by signing the pre-written consent form. Minors (15 to 17 years old) who agreed to participate assented by signing the minor's consent form, and their guardians also had to confirm their support by appending their signatures or thumb prints. Individuals who consented to participate had the option of either responding to the interview only, providing a blood sample only, or providing both.

The survey used a questionnaire to collect data on knowledge of HIV prevention, HIV testing history, marriage and sexual activity, and circumcision. HIV status was determined using HIV serology on dried blood spots obtained from participants through a finger prick using Determine^® ^HIV-1/HIV-2 (Abbott) and Uni-Gold™ Test kits, according to manufacturer's instructions. By design, participants were not allowed to know their HIV status results from the blood sample provided for the study. Those who wanted to know their status were provided standard pre-test counseling, testing, and post-test counselling at a Voluntary Counselling and Testing Centre. Core variables from the NUHDSS database were linked anonymously to the survey and serodata results using a linking ID.

### Data analysis

Descriptive and multivariate logistic regression analyses were carried out to describe participation by sociodemographic characteristics and to assess determinants of sampled individuals being contacted and determinants for agreeing to provide a blood sample for HIV testing among those contacted.

To facilitate assessment of potential bias in the HIV seroprevalence estimates, the analysis was carried out using multivariate multiple imputation techniques using a set of variables where data were available for the various interview outcome categories. Irrespective of the interview outcome, all sampled individuals had a minimum set of sociodemographic data derived from the NUHDSS database. Using multivariate imputation methods (Multivariate Imputation with Chained Equations-MICE) for missing data as described by van Buuren et al [21] and implemented in Stata software by Royston [22], HIV prevalence among nonresponders was estimated using a common set of variables, including age, gender, residence, ethnicity, marital status, educational attainment, mobility index, and socioeconomic status (using a wealth index constructed from household items). For the category of individuals who were interviewed but not tested, an additional set of variables from the survey questionnaire was included in the imputation models for this subgroup. We used a logistic model to assess how predictive of HIV the sociodemographic characteristics were. This gave a pseudo R squared of about 11%. The model improved to 32% when additional variables from the survey (on HIV knowledge and attitudes and sexual behavior) were added, including: male circumcision; age at first sex; number of partners in last 12 months; high-risk sex; stigma indicator *-"keep it a secret if family member is HIV positive"*; condom use at last sex; and ever tested for HIV. The mobility index was derived from NUHDSS records on each individual's movement episodes within and out of the slum per unit time as described later. For this paper, nonresponse to HIV testing refers to the following categories: i) No contact made with participant, ii) participant contacted but refused to give a blood sample and interview, and iii) participant consented to provide the interview but refused to give a blood sample. Results are presented separately for each gender because descriptive results showed significant differences in contact rates by gender. The different participant response categories and available variables are outlined in Table 1.

**Table 1:** Participant response categories considered during imputation

Participant category	Participant survey outcome	Variables available	Numbers (%)
1	Interviewed and tested or tested only	HIV status, socio-demographic, mobility index, HIV knowledge and attitudes, and sexual behaviour variables	2721 (57.1)

2	Interviewed but not tested	Socio-demographic, mobility index, HIV knowledge and attitudes, and sexual behaviour variables	573 (12.0)

3	Refused both interview and testing	Socio-demographic & mobility index data only	203 (4.3)

4	Not contacted at all	Socio-demographic & mobility index data only	1270 (26.6)

Total	-	-	4767 (100)

As pointed out by Marston et al [3], mobility is an important risk factor for HIV and, whenever possible, should be factored into the adjustments. Mobility data were available for all individuals as they were derived from the demographic surveillance database. The mobility index was derived from a count of movement episodes of participants within or out of the surveillance area per unit time. An individual was considered to be highly mobile if she or he had at least one or more episodes of change of residence per year or at least one out-migration and return episode to the surveillance area in two years.

The missing HIV status for those in category 2 was imputed against category 1, which had HIV status data, sociodemographic variables, and survey data. Missing HIV status data in categories 3 and 4 were imputed separately against category 1 using sociodemographic variables and mobility index. Multiple imputation was carried out using Stata version 10 statistical software using a user-written program called ice [22,23]. The ice program does not assume multivariate joint distribution as do other multivariate approaches of handling missing data. This makes it flexible and more appealing to use. Imputations for HIV status data were carried out separately for each of the three participant categories that had no HIV status data and by gender. For each category, using the multiple imputation program, we created 10 multiple datasets *(*5-10 multiple copies are recommended) with missing data inserted as predicted by the variables in the model. The ice command automatically creates and combines the multiple imputed data files to get a single data file for a given category. From the combined file, prevalence estimates and corresponding confidence intervals for proportions were derived. The overall corrected HIV prevalence estimate (observed and imputed) was taken to be a weighted average of the imputed and observed prevalences, and an overall confidence interval for the resultant prevalence was also derived.

## Results

Overall, approximately 73.4% of the sampled individuals were successfully contacted. Out of the 3,497 individuals who were contacted, 2,721 (57.1% of the overall sample) agreed to provide a blood sample. The percentage of those who were contacted and agreed to be tested was 77.8%. Table 2 shows the percentage distribution of those contacted, those who agreed to be tested, and HIV prevalence by sociodemographic characteristics. The chi-square statistic and corresponding p-values show that there were statistically significant differences between those contacted and those not contacted by age, gender, residence, ethnicity, mobility, and wealth status (p-value < 0.05). Proportionately more women and residents of Korogocho slums were contacted. Individuals in the lowest wealth quintile, the highly mobile, and those never married were less likely to be contacted as opposed to those in the wealthier quintiles, the less mobile, and those who had ever been married. The percentage distribution of formal education attainment between those contacted and those not contacted was not significantly different.

**Table 2:** Percentage distribution of participants who were successfully contacted, those who accepted to test and HIV test result by socio-demographic characteristics

Variables	Total Number	Contacted		Number Contacted	Accepted to test		HIV prevalence among tested	
					
		Percent (%)	***X***^**2 **^**(P-value)**		Percent (%)	***X***^**2 **^**(P-value)**	Percent positive	***X***^**2 **^**(P-value)**
Age group								
< 20 yrs	611	77.7		475	87.0		5.1	
20-24 yrs	1,120	71.3		798	79.7		7.2	
25-29 yrs	1,028	71.4	21.1 (0.004)	734	76.8	39.3 (< 0.001)	12.1	59.9 (< 0.001)
30-34 yrs	750	75.6		567	72.1		13.7	
35-39 yrs	555	75.5		419	74.9		17.2	
40-44 yrs	368	73.1		269	76.2		13.7	
45-49 yrs	249	73.5		183	78.1		21.0	
50-54 yrs	86	60.5		52	71.2		24.3	
Gender								
Female	2,931	75.7	20.9 (< 0.001)	2,218	77.2	1.2 (0.279)	12.4	4.3 (0.039)
Male	1,836	69.7		1,279	78.8		9.8	
Residence								
Korogocho	2,435	76.6	26.6 (< 0.001)	1,865	85.3	130.1 (< 0.001)	13.9	22.2 (< 0.001)
Viwandani	2,332	70.0		1,632	69.2		8.1	
Ethnicity								
Kikuyu	1,504	71.5		1,076	76.9		8.2	
Luhya	693	76.5	13.7 (0.008)	530	84.7	97.9 (< 0.001)	11.6	83.7 (< 0.001)
Luo	795	75.4		599	87.0		22.7	
Kamba	1,092	70.8		773	66.9		8.3	
Others	683	76.0		519	78.4		7.6	
Education								
No schooling	179	76.0		136	75.7		23.3	
Primary	3,029	73.6	1.6 (0.671)	2,228	79.0	5.4 (0.143)	12.1	21.1 (< 0.001)
Secondary/higher	1,474	72.9		1,048	75.7		8.7	
Don't know	85	69.4		59	74.6		9.1	
Wealth Index								
Poorest 20%	889	57.0		507	77.3		13.3	
2^nd^	958	71.5	185.0 (< 0.001)	685	75.5	3.5 (0.473)	13.4	6.4 (0.171)
3^rd^	991	75.3		746	78.4		11.3	
4^th^	953	78.5		748	79.4		11.3	
Richest 20%	976	83.1		811	78.1		9.2	
Marital status								
Married	2,919	77.2		2,254	75.3		11.6	
Divorce/widowed	437	80.1	220.0 (< 0.001)	350	82.0	28.3 (< 0.001)	26.5	90.3 (< 0.001)
Never married	1,335	66.3		885	82.8		5.5	
Not known	76	10.5		8	50.0		0.0	
Mobility								
Not highly mobile	4358	76.7	155.6 (< 0.001)	3115	77.3	3.7 (0.054)	11.8	2.2 (0.142)
Highly mobile	409	54.2		382	81.7		9.0	
								
Total	4767	73.4		3497	77.8		11.5	(10.3-12.7)§

§ 95% confidence interval

With regard to agreeing to be tested, higher proportions of younger individuals, residents of Korogocho, and members of the Luhya and Luo ethnic backgrounds accepted the test than their counterparts. There were no significant differences between those who accepted to test and those who refused by gender, educational attainment, and wealth status. The distribution of HIV prevalence by age, ethnicity, slum of residency, educational attainment, and marital status showed significant variation across several variables. Individuals below 20 years of age had the lowest prevalence but one of the highest participation rates, while men had lower participation rates and lower HIV prevalence. On the other hand, residents of Korogocho had higher participation rates and higher HIV prevalence. The Luo and Luhya ethnic groups and the widowed/divorced had higher participation rates and corresponding higher HIV prevalence than their counterparts.

### Multivariate Analysis

Table 3 provides odds ratios derived from a logistic model for sampled individuals being successfully contacted by gender, controlling for a set of sociodemographic characteristics. With regard to age, results show a general trend for both men and women. The older an individual was, the less likely she or he was to be contacted, although this was not significant for all categories. There was no significant association between being contacted and educational attainment. Women from the Luhya tribe and men from Luhya and Kamba tribes were significantly more likely to be contacted compared to the Kikuyu ethnic group.

**Table 3:** Logistic regression model results showing odds ratios of being successfully contacted by gender controlling for socio-demographic characteristics

Variables	Women			Men		
	Number	Odds Ratio [95% CI]	P value	Number	Odds Ratio [95% CI]	P value
Age category						
Less than 20 yrs	431	1.52[1.11-2.07]	0.009	180	1.83[1.17-2.84]	0.008
20-24 yrs	819	1.00		301	1.00	
25-29 yrs	657	0.79[0.61-1.01]	0.063	371	0.94[0.65-1.35]	0.725
30-34 yrs	422	0.82[0.61-1.12]	0.214	328	0.90[0.59-1.35]	0.600
35-39 yrs	304	0.76[0.54-1.07]	0.121	251	0.74[0.47-1.15]	0.178
40-44 yrs	194	0.76[0.50-1.15]	0.191	174	0.58[0.36-0.93]	0.024
45-49 yrs	104	0.67[0.39-1.16]	0.153	145	0.61[0.37-1.02]	0.062
50-54 yrs	--	--	--	86	0.40[0.23-0.71]	0.002
Ethnicity						
Kikuyu	967	1.00		537	1.00	
Luhya	439	1.36[1.02-1.82]	0.039	254	1.43[1.00-2.05]	0.049
Luo	482	1.29[0.97-1.73]	0.080	313	1.16[0.83-1.61]	0.385
Kamba	612	1.06[0.82-1.37]	0.659	480	1.58[1.16-2.15]	0.004
Others	431	1.31[0.96-1.78]	0.083	252	1.29[0.91-1.84]	0.158
Wealth Index						
Poorest	466	1.00		423	1.00	
2^nd^	531	1.62[1.22-2.16]	0.001	427	1.52[1.12-2.06]	0.007
3^rd^	620	1.78[1.35-2.36]	< 0.001	371	1.69[1.23-2.32]	0.001
4^th^	648	2.07[1.56-2.77]	< 0.001	305	1.82[1.29-2.56]	0.001
Richest	666	2.55[1.89-3.44]	< 0.001	310	2.97[2.04-4.33]	< 0.001
Education level						
Never schooled	136	0.94[0.58-1.54]	0.813	43	0.56[0.28-1.10]	0.093
Primary	1,985	1.00		1,044	1.00	
Secondary/higher	753	1.03[0.83-1.27]	0.815	721	1.12[0.89-1.41]	0.336
Don't Know	57	0.69[0.37-1.29]	0.242	28	2.06[0.78-5.47]	0.146
Residence						
Korogocho	1,542	1.00		893	1.00	
Viwandani	1,389	0.72[0.58-0.88]	0.001	943	0.82[0.64-1.06]	0.131
Mobility Index						
Not highly mobile	2,450	1.00		1,612	1.00	
Highly mobile	481	0.41[0.32-0.51]	< 0.001	224	0.39[0.28-0.54]	< 0.001
Marital status						
Married	1,774	1.00		1,145	1.00	
Divorced/widowed	339	1.11[0.81-1.51]	0.526	98	1.73[1.00-2.98]	0.050
Never married	779	0.47[0.37-0.59]	< 0.001	556	0.43[0.32-0.59]	< 0.001
Not Known	39	0.04[0.01-0.11]	< 0.001	37	0.06[0.02-0.18]	< 0.001
Total	2,931			1,836		

For both sexes, individuals from the wealthiest households were more than 2.5 times more likely to be contacted compared to their poorest counterparts. Women from Viwandani were less likely to be contacted compared to women from Korogocho, but there were no significant differences among men. Women and men classified as highly mobile were less likely to be contacted compared to those classified as less mobile. Women and men who had never been married were significantly less likely to be contacted compared to their married counterparts.

Table 4 shows the odds ratios for agreeing to provide blood samples once contacted by gender. Women aged 30 to 34 were significantly less likely to accept being tested compared to those aged 20 to 24, while teenage males were up to more than three times as likely to accept testing compared to those aged 20 to 24. Educational level, wealth status, and being highly mobile were not significantly associated with accepting to be tested. For both sexes, individuals from the Luhya and Luo ethnic backgrounds were more likely to accept being tested compared to their Kikuyu counterparts. Widowed or divorced men were about 2.3 times more likely to accept testing than currently married men, while for women, the never married were about 1.4 times more likely to accept HIV testing compared to their married counterparts. Residents of Viwandani slum were generally less likely to accept testing compared to residents of Korogocho slum.

**Table 4:** Logistic regression model results showing odds ratios of accepting to be tested by gender controlling for socio-demographic characteristics

Variables	Women			Men		
		
	Number	Odds Ratio[CI]	P value	Number	Odds Ratio[CI]	P value
Age category						
Less than 20 yrs	339	1.02[0.69-1.51]	0.903	136	3.25[1.43-7.40]	0.005
20-24 yrs	608	1.00		190	1.00	
25-29 yrs	477	1.02[0.75-1.38]	0.915	257	0.85[0.51-1.42]	0.537
30-34 yrs	325	0.63[0.46-0.88]	0.007	242	0.85[0.50-1.47]	0.573
35-39 yrs	235	0.73[0.50-1.07]	0.106	184	0.91[0.50-1.63]	0.739
40-44 yrs	153	0.90[0.57-1.44]	0.669	116	0.71[0.38-1.35]	0.299
45-49 yrs	81	0.84[0.45-1.56]	0.583	102	0.93[0.47-1.82]	0.824
50-54 yrs	--	--	--	52	0.63[0.29-1.37]	0.240
Ethnicity						
Kikuyu	723	1.00		353	1.00	
Luhya	344	1.72[1.22-2.45]	0.002	186	1.96[1.18-3.25]	0.009
Luo	382	2.04[1.41-2.96]	< 0.001	217	1.48[0.94-2.36]	0.094
Kamba	428	0.80[0.60-1.06]	0.117	345	0.90[0.61-1.31]	0.567
Others	341	1.18[0.84-1.64]	0.338	178	1.52[0.94-2.48]	0.091
Wealth Index						
Poorest	276	1.00		231	1.00	
2^nd^	386	1.16[0.80-1.69]	0.438	299	0.90[0.58-1.40]	0.630
3^rd^	477	1.19[0.82-1.72]	0.358	269	0.90[0.57-1.42]	0.648
4^th^	523	1.26[0.87-1.82]	0.219	225	0.93[0.57-1.51]	0.775
Richest	556	1.14[0.79-1.65]	0.482	255	0.89[0.55-1.44]	0.629
Education level						
Never schooled	111	0.61[0.37-1.00]	0.050	25	1.05[0.33-3.33]	0.936
Primary	1,507	1.00		721	1.00	
Secondary/higher	562	1.19[0.93-1.52]	0.179	512	0.87[0.65-1.17]	0.365
Don't Know	38	0.76[0.34-1.66]	0.489	21	0.75[0.27-2.04]	0.570
Residence						
Korogocho	1,231	1.00		634	1.00	
Viwandani	987	0.39[0.30-0.49]	< 0.001	645	0.66[0.47-0.91]	0.013
Mobility Index						
Not highly mobile	1,941	1.00		1,174	1.00	
Highly mobile	277	1.22[0.87-1.72]	0.246	105	1.63[0.90-2.95]	0.108
Marital status						
Married	1,405	1.00		849	1.00	
Divorced/widowed	271	1.35[0.95-1.91]	0.095	79	2.26[1.09-4.69]	0.029
Never married	538	1.39[1.03-1.89]	0.031	347	1.02[0.66-1.56]	0.938
Not Known	4	0.41[0.05-3.32]	0.404	4	0.37[0.05-2.73]	0.330
Total	2,218			1,279		

Table 5 shows observed and adjusted prevalence of HIV for men and women separately and the overall combined estimates. In all interview and test outcome categories, the observed and imputed prevalences for HIV were higher among women than men. The imputed prevalences for women in all categories were lower than the observed. Women who were not tested had an imputed prevalence lower than the observed prevalence by 6%. The overall corrected prevalence among women was lower than the observed by 3%. Males who were not tested had an imputed prevalence higher than the observed prevalence by 2%, while the overall corrected HIV prevalence for men was higher than the observed by 1%. The overall adjusted prevalence of HIV for both sexes was lower than the observed by about 2%.

**Table 5:** Observed, imputed and overall adjusted prevalence of HIV

		Females		Males		Total
						
Interview and testing status	N	Prevalence [95% CI]	N	Prevalence [95% CI]	Total	Prevalence [95% CI]
						
Interviewed & Tested-*Observed prevalence (a)*	1713	12.43 [10.87-14.00]	1008	09.82 [07.98-11.66]	2721	11.47 [10.27-12.67]
						
Interviewed only-*imputed prevalence (b)*	363	11.93 [10.89-13.03]	210	8.95 [7.77-10.26]	573	10.84 [10.04-11.67]
						
Refused both-*imputed prevalence (c)*	142	10.07 [8.55-11.75]	61	9.34 [7.15-11.94]	203	9.85 [8.59-11.23]
						
No contact made-*imputed prevalence (d)*	713	11.78 [11.04-12.55]	557	10.43 [9.64-11.26]	1270	11.19 [10.65-11.75]
						
Imputed prevalence all non-response *-imputed prevalence-b,c & d (e)*	1218	11.63 [9.86-13.46]	828	9.97 [7.97-12.07]	2046	10.96 [9.6-12.30]
						
Overall, corrected prevalence-*adjusted (f)*	2931	12.10 [10.93-13.29]	1836	9.89 [8.54-11.28]	4767	11.25 [10.34-12.14]
						
*Ratio (e/a)*		0.94		1.02		0.96
*Ratio (f/a)*		0.97		1.01		0.98

## Discussion

This paper explored nonresponse to HIV testing in a survey and its impact on HIV prevalence estimates in informal settlements with a relatively mobile and young population. Nonresponse to HIV testing in this study (43%) was quite high compared to other community-based HIV testing surveys [1-3]. Absenteeism contributed 62%, while refusals accounted for 38% of nonresponse. At the time of designing the survey, an estimated nonresponse rate of 40% was factored into the sample size estimation, based on what has been reported elsewhere and the attrition rates in the NUHDSS.

Bivariate and multivariate assessments of responders and nonresponders showed that there were statistically significant differences between the two groups, justifying the need to assess the extent to which the observed differences could have affected the overall estimate of HIV prevalence in this population. Age, socio-economic status, residence, and mobility index were found to be good predictors of whether an individual was likely to be successfully contacted or not. Older people were not only less likely to be contacted, but they were also less likely to accept HIV testing. This finding was a bit surprising. One would have expected younger adults to be more mobile and less inclined to spare their time to participate in the survey. However, it should be noted that economic survival in the informal settlements relies on a cash economy dominated by informal employment. It might be the case that older people (up to 49 years for women and up to 54 years for men) have more demanding family responsibilities and as such are likely to be away from home fending for their families. Similarly, residents of Viwandani were less likely to be found at home, and if found, they were less inclined to participate. This finding is in line with our expectation. Viwandani slum is predominately inhabited by young adults, with smaller families and more educated residents who are more likely to be working in the nearby industrial estate, hence the higher likelihood of not being found at home.

Members of two of the ethnic communities with the highest HIV prevalence in Kenya, the Luo and Luhya, [16] were more likely to be contacted compared to their Kikuyu counterparts, and furthermore, they were also more likely to accept testing. It is hard to find an explanation for this observation. As expected, the mobility index predicted the likelihood of being found at home but not necessarily that of accepting to participate. If all these dynamics were examined in isolation, it would be hard to predict the likely overall impact the differential participation would have on HIV estimates. The odds of participation in the survey were not consistently higher among subgroups that are characteristically known to have higher or lower HIV prevalence such as age, gender, ethnicity, marital status, and socio-economic status. Thus from descriptive results, it is difficult to guess the overall direction the results would be biased, if at all, given that both participation rates and observed HIV prevalence varied in various directions by the key sociodemographic variables.

In the final model of multiple imputations, the overall effect on the estimates was small, showing that contrary to our expectation, HIV prevalence appears to have been overestimated by about 2%. Imputed estimates among females were consistently lower than the observed prevalence. It is important to note that all observed estimates lie within the confidence limits of the adjusted estimates, indicating that differences are small in spite of the significant differences in participation rates by sociodemographic characteristics as noted in Tables 2, 3, and 4. The high nonresponse rate observed in this study notwithstanding, results show that sound estimates can be obtained in a community-based HIV seroprevalence survey in a similar setting. The observed bias in this study is minimal but has a gender component, with a tendency to overestimate prevalence among women and underestimate it among males.

Future work in similar settings should take into consideration a number of issues. The informal nature of the housing makes listing of households extremely difficult. In the absence of a dedicated registration and monitoring system such as the demographic surveillance system, having an updated sampling frame is nearly impossible. Ways around this challenge should be carefully considered from the start. Although this study has found that the impact of nonresponse on overall estimates was minimal, it is prudent to adequately sample the population, factoring in nonresponse rates based on attrition rates where available. Extra efforts to reach hard-to-contact individuals must be considered while planning the study, especially in terms of duration of the study and adequacy of field staff.

### Limitations

Although the NUHDSS provided background characteristics for all sampled individuals, including nonresponders, the set of variables was rather limited for predicting the risk of HIV infection. It is possible that nonresponders were significantly and systematically different from responders on characteristics other than those used in the adjustments. The multiple imputations method used also assumes that data missing are missing at random (MAR). In reality, this might not be the case, and the predictions may not be as good. Mobility, as pointed out in other studies, is a key predictor of HIV infection, yet the way the mobility index was measured falls short of capturing short-term movements, such as absences of days or weeks, as happens with long-distance truck drivers. Movements involving short durations of absence might actually be more important in exposing individuals to the risk of HIV than movements involving longer periods of absence. Non-return migration can also result in underestimation of HIV prevalence, especially if the reason for out-migration is associated with poor health, as is the case with terminally ill HIV/AIDS patients. One study noted markedly high HIV/AIDS-related death rates among rural returnees in South Africa [24], indicating that a significant proportion of rural return migrants were HIV positive. HIV prevalence in the origin population could be affected (lowered) as a result of selective out-migration of infected individuals.

## Conclusions

The estimate of HIV prevalence in slums is higher than that reported for Nairobi province, with women being disproportionately affected. Nonresponse resulted in minimal overestimates of HIV prevalence overall. We also infer that it is possible to obtain reliable results even in a relatively mobile population under surveillance as long as proper considerations are made at the survey design and implementation stages.

## Competing interests

The authors declare that they have no competing interests.

## Authors' contributions

AKZ took the lead in preparing the manuscript and participated in the study design, implementation, data management and analysis, and manuscript write-up. NJM was the principal investigator and participated in proposal development, project implementation, supervision, and manuscript preparation. MM participated in proposal development, project implementation, supervision of testing of the blood samples in the laboratory, and manuscript preparation. EZ participated in proposal development, advised on aspects of implementation, and participated in manuscript preparation. JK participated in proposal development, project implementation, field supervision, and manuscript preparation. VO participated in proposal development, project implementation, testing of the blood samples in the laboratory and manuscript preparation. SK participated in proposal development, project implementation, testing of the blood samples in the laboratory, and manuscript preparation. ACE participated in proposal development, project implementation, supervision, and overall project management. He also participated in manuscript preparation. All authors read and approved the final manuscript.
